# Analysis of early and treatment related deaths among children and adolescents with acute myeloid leukemia in Poland: 2005–2023

**DOI:** 10.3389/fped.2024.1482720

**Published:** 2024-10-17

**Authors:** Katarzyna Pawińska-Wąsikowska, Małgorzata Czogała, Karolina Bukowska-Strakova, Marta Surman, Monika Rygielska, Teofila Książek, Beata Sadowska, Agnieszka Pac, Jolanta Skalska-Sadowska, Magdalena Samborska, Jacek Wachowiak, Małgorzata Ciebiera, Radosław Chaber, Renata Tomaszewska, Tomasz Szczepański, Karolina Zielezińska, Tomasz Urasiński, Anna Rodziewicz-Konarska, Krzysztof Kałwak, Marta Kozłowska, Ninela Irga-Jaworska, Barbara Sikorska-Fic, Bartosz Chyżyński, Paweł Łaguna, Katarzyna Muszyńska-Rosłan, Maryna Krawczuk-Rybak, Paulina Deleszkiewicz, Katarzyna Drabko, Katarzyna Bobeff, Wojciech Młynarski, Agnieszka Chodała-Grzywacz, Grażyna Karolczyk, Katarzyna Mycko, Wanda Badowska, Natalia Bartoszewicz, Jan Styczyński, Katarzyna Machnik, Weronika Stolpa, Agnieszka Mizia-Malarz, Walentyna Balwierz, Szymon Skoczeń

**Affiliations:** ^1^ Department of Pediatric Oncology and Hematology, Institute of Pediatrics, Jagiellonian University Medical College, Krakow, Poland; ^2^ Department of Pediatric Oncology and Hematology, University Children Hospital of Krakow, Krakow, Poland; ^3^ Department of Clinical Immunology, Institute of Pediatrics, Jagiellonian University Medical College, Krakow, Poland; ^4^ Department of Pediatric Oncology and Hematology, Hematology Laboratory, University Children’s Hospital, Krakow, Poland; ^5^ Department of Molecular Genetics, Institute of Pediatrics, Jagiellonian University Medical College, Krakow, Poland; ^6^ Department of Pediatric Oncology and Hematology, Cytogenetics and Molecular Genetics Laboratory, University Children’s Hospital, Krakow, Poland; ^7^ Department of Epidemiology and Preventive Medicine, Faculty of Medicine, Jagiellonian University Medical College, Kraków, Poland; ^8^ Department of Pediatric Oncology, Hematology and Transplantology, Poznan University of Medical Sciences, Poznan, Poland; ^9^ Department of Pediatric Oncohematology, Clinical Province Hospital of Rzeszow, Rzeszow, Poland; ^10^ Department of Pediatrics, Institute of Medical Sciences, Medical College, University of Rzeszow, Rzeszow, Poland; ^11^ Department of Pediatric Hematology and Oncology, Zabrze, Medical University of Silesia, Katowice, Poland; ^12^ Department of Pediatrics, Hemato-Oncology and Gastroenterology, Pomeranian Medical University in Szczecin, Szczecin, Poland; ^13^ Clinical Department of Pediatric Bone Marrow Transplantation, Oncology and Hematology, Wroclaw Medical University, Wroclaw, Poland; ^14^ Department of Pediatrics, Hematology and Oncology, Medical University of Gdansk, Gdansk, Poland; ^15^ Department of Oncology, Pediatric Hematology, Transplantology and Pediatrics, Medical University of Warsaw, Warsaw, Poland; ^16^ Departament of Pediatrics, Oncology and Hematology Medical University of Bialystok, Bialystok, Poland; ^17^ Department of Pediatric Hematology, Oncology and Transplantology, Medical University of Lublin, Lublin, Poland; ^18^ Department of Pediatrics, Oncology and Hematology, Medical University of Lodz, Lodz, Poland; ^19^ Department of Pediatric Hematology and Oncology, Regional Polyclinic Hospital in Kielce, Kielce, Poland; ^20^ Department of Pediatrics and Hematology and Oncology, Province Children’s Hospital, Olsztyn, Poland; ^21^ Department of Pediatric Hematology and Oncology, Collegium Medicum, Nicolaus Copernicus University Torun, Bydgoszcz, Poland; ^22^ Department of Pediatrics, Hematology and Oncology, City Hospital, Chorzow, Poland; ^23^ Department of Pediatrics, Upper Silesia Children’s Care Health Centre, Medical University of Silesia, Katowice, Poland

**Keywords:** acute myeloid leukemia, early death, treatment related death, children, adolescents

## Abstract

**Background:**

A personalised approach to the treatment of acute myeloid leukemia (AML) in children and adolescents, as well as the development of supportive therapies, has significantly improved survival. Despite this, some patients still die before starting treatment or in an early phase of therapy before achieving remission. The study analysed the frequency, clinical features and risk factors for early deaths (ED) and treatment related deaths (TRD) of children and adolescents with AML.

**Methods:**

From January 2005 to November 2023, 646 children with AML treated in the centers of the Polish Pediatric Leukemia and Lymphoma Study Group according to three subsequent therapeutic protocols were evaluated: AML-BFM 2004 Interim (385 children), AML-BFM 2012 Registry (131 children) and AML-BFM 2019 (130 children).

**Results:**

Out of 646 children, early death occurred in 30 children, including 15 girls. The median age was 10.7 years (1 day to 18 years). More than half of the patients (53%) were diagnosed with acute myelomonocytic leukemia (M5) and 13% with acute promyelocytic leukemia (M3). The ED rate for the three consecutive AML-BFM protocols was 4.9% vs. 5.3% vs. 3.1%, respectively. In 19 patients, death occurred before the 15th day of treatment, in 11 between the 15th and 42nd day. The most common cause of death before the 15th day (ED15) was leukostasis and bleeding, whereas between the 15th and 42nd day (ED15-42), infections, mainly bacterial sepsis. A significant association was found between ED15 and high leukocyte count (>10 × 10^9^/L), M3 leukemia (*p* < 0.001), and ED15-42 and age <1 year (*p* = 0.029). In the univariate analysis only initial high leukocyte count >100 × 10^9^/L, was a significant predictor of early death. The overall TRD for the entire study period was 3.4%. The main cause of death were infections, mainly bacterial sepsis (10 children out of 22, 45.4%).

**Conclusions:**

Hyperleukocytosis remains significant factor of early mortality in patients with AML, despite the introduction of various cytoreductive methods. Infections are still the main cause of treatment related deaths. A more individualized approach by using new targeted drugs may be the therapeutic option of choice in the future.

## 1 Introduction

A personalized approach to the treatment of acute myeloid leukemia (AML) in children and adolescents has significantly improved treatment outcomes. Tailoring the intensity of therapy based on the presence of specific genetic abnormalities in leukemic blasts and the measurable residual disease (MRD) rate after induction chemotherapy significantly improved survival not only by reducing the rate of relapse, deaths due to disease progression, but also deaths due to treatment toxicity (1–4). The development of modern therapies in pediatric AML is without a doubt a success story in improving survival. However, some children still experience treatment failure in different stages of therapy. With the development of optimal blocks of induction chemotherapy and supportive therapy, the incidence of early death (ED) and treatment-related death (TRD) has decreased in recent decades (5–12).

The results of three consecutive therapy protocols used by the Polish Pediatric Leukemia and Lymphoma Study Group (PPLLSG) since 1983–2004 showed a substantial improvement in survival in pediatric AML. The incidence of early deaths (before day 42 of therapy) in the first period, years 1983–1993, when the AML-PPLLSG 83 protocol was used, was 24%, in the second period, years 1994–1997 (AML-PPLLSG 94 protocol) decreased 14%, and in the third period, years 1998–2003 (AML-PPLLSG 98 protocol) another drop in ED to 5% was reported (6).

There are limited studies on predictors of ED and TRD in children and adults, and the results are contradictory for some parameters, such as initial leukocyte count (11–15). Without a doubt, knowledge of early mortality predictors may help identify the most vulnerable population among patients with AML who need special attention since the beginning of therapy, as well as improve supportive care strategies in children from the first days of therapy. The aim of the presented study was to characterise the frequency, clinical characteristics and risk factors for early death and treatment-related mortality in children and adolescents with acute myeloid leukemia between 2005 and 2023, when PPLLSG used three consecutive treatment protocols.

## 2 Materials and methods

### 2.1 Patients

We retrospectively analyzed the national AML database of the PPLLSG. Since January 2005–November 2023, 646 children with acute myeloid leukemia were treated in the centers of the PPLLSG according to three subsequent therapeutic protocols: in the first period, 2004–2015: protocol AML-BFM 2004 Interim (385 children), in the second period, 2015–2019: protocol AML-BFM 2012 Registry (131 children) and in the third period, 2019–2023: AML-BFM 2019 Recommendations (130 children). Patients with mixed immunophenotype leukemia, secondary myeloid leukemia, myeloid sarcoma, myeloid leukemia due to myelodysplastic syndrome, and myeloid leukemias in Down syndrome were excluded from the analysis. Baseline data such as patient characteristics, therapy details, and treatment outcomes, including cause of death, were collected from the PPLLSG AML database. In case of missing specific data, the medical records of eligible patients were reviewed on the PPLLSG site by dedicated study members. The diagnosis of AML and its subtype was made according to the French-American-British (FAB) classification, confirmed by multiparametric flow cytometry and molecular tests for AML characteristics genetic rearrangements. All diagnoses were confirmed by the central laboratory. The study protocol has been carried out in accordance with The Ethics Code of the World Medical Association (Declaration of Helsinki) for experiments involving humans and approved by the Ethics Committee of Jagiellonian University (protocol code—118.0043.1.154.2024, date of approval 13.05.2024).

### 2.2 Definitions

Early death was defined as the death that occurred between the beginning of therapy and 42 day of treatment (ED). Very early death was defined as a death between day 1 and 14 of therapy (two first weeks of therapy), ED15. Death between day 15 and 42 of therapy was classified as ED15-42. Death of the patient in complete remission (CR) or absence of progression was defined as treatment-related death (TRD). In patients who died before day 15 or before 28 in the second and third period of the study, the response was undeterminable.

The definition of complete remission (CR) was described as less than 5% of the blasts in the bone marrow, with an absolute neutrophil count >1 × 10^9^/L, platelets 100 × 10^9^/L in peripheral blood, and without signs of extramedullary involvement.

Hyperleukocytosis was defined as the initial white blood count was above 100 × 10^9^/L.

The following genetic abnormalities of leukemic cells were considered as favorable genetics (abnormal genetics with favorable prognosis) in the study: t(8;21)(q22;q22)/RUNX1::RUNX1T, inv(16)(p13.1q22) or t(16;16)(p13.1;q22)/CBFB::MYH11; and t(15;17)/PML::RARA; whereas unfavorable genetics (abnormal genetics with unfavorable prognosis) were considered: monosomy 7, t(6;9)(p23;q34)/DEK::NUP214, t(9;22)(q34;q11.2)/BCR::ABL1; FLT3-ITD mutation, 11q23/KMT2A abnormalities, i.e., t(6;11)(q27;q23)/KMT2A::AFDN; t(10;11)(p12;q23)/KMT2A::MLLT10; t(8;16)(p11.p13)/KATA6::CREBBP or complex karyotype. Complex karyotype was diagnosed as three or more aberrations, including at least one structural aberration, without favorable genetics and without KMT2A rearrangement in leukemic cells. The presence of genetic rearrangements that were not classified as favorable or unfavorable genetics was considered as “other genetic abnormalities.” A normal karyotype was defined as the absence of clonal aberrations in the metaphases of bone marrow aspirates at the time of diagnosis.

We did not analyze deaths in relapsed and refractory patients unless deaths occurred before 42 days of therapy in the latter. Deaths due to allogeneic-hematopoietic stem cell transplantation (allo-HSCT) were registered, however, more specific information on SCT was beyond the scope of the presented study.

To identify risk factors for ED in patients with AML, we analyzed age, gender, French-American-British (FAB) type, treatment risk group, leukocyte count at diagnosis, genetic rearrangements, central nervous system (CNS) involvement, and ECOG status. CNS disease was characterized as more than 5 white blood cells in 1 mm^3^ and unequivocal evidence of leukemic blasts in a nonbloody tap, and/or clinical symptoms or signs of involvement (seizures, cranial nerve palsy, increased symptoms of cranial pressure symptoms), and/or radiological evidence of leukemic infiltration of the CNS in imagine studies.

The Eastern Cooperative Oncology Group (ECOG) performance scale was used for assessment the patient's status at diagnosis. Patients who died before the implementation of therapy were excluded for the final analysis.

### 2.3 Treatment

All three subsequent therapeutic protocols: AML-BFM 2004 Interim, AML-BFM 2012 Registry and AML-BFM 2019 Recommendations were based on anthracycline, cytarabine and etoposide backbone. In the AML-BFM 2004 Interim protocol, after one induction block, patients were classified into to standard (SRG) or to high risk group (HRG) based on response to therapy. In case of FLT3-ITD mutation patients were reclassified as HRG.

An additional induction block was administered to patients classified as HRG. All patients received three consolidation chemotherapy blocks, followed by maintenance therapy with CNS radiotherapy in SRG patients. HRG patients received allogeneic hematopoietic stem cell transplantation (allo-HSCT) from a matched sibling donor if available. Nonresponders, patients with >5% of blasts after the second induction received allo-HSCT from a matched unrelated donor. In the AML-BFM 2012 Registry and AML-BFM 2019 Recommendations protocols, after two courses of induction chemotherapy patients were stratified into one of the risk groups, standard, intermediate (IRG), or high, according to genetic abnormalities of leukemic cells and response to therapy. Further consolidation treatment consisted of two or three chemotherapy blocks, depending on the risk group. Patients stratified into the HRG received allo-HSCT, while non-HR patients received 1 year of maintenance therapy. Children with a leukocyte number above 50 × 10^9^/L started with cytoreductive treatment with cytarabine. The details of therapy and stratification to risk groups in the protocols are shown in Supplementary Material Figure S1, Table S1.

Therapy for patients with APL: In the first period when AML-BFM 2004 Interim was used, it consisted of four intensive chemotherapy cycles (AIE: cytarabine, idarubicine, etoposide, AI: cytarabine, idarubicine, haM: high-dose cytarabine, mitoxantron, HAE: high-dose cytarabine, etoposide; additional intrathecal cytarabine in every cycle) and maintenance therapy (6-thioguanine, cytarabine) for 1 year. All patients received all-trans retinoid acid (ATRA) concomitant with chemotherapy in 14-day cycles (16). In second and third period, when AML-BFM 2012 Registry protocol, and subsequently AML-BFM 2019 Recommendations were used patients with APL were classified into two groups according to initial number of white blood cells (WBC). Patients in the standard risk group with an initial WBC <10 × 10^9^/L were treated with the ATRA and arsenic acid (ATO) regimen. Other patients were classified into the high risk group (more than 10 × 10^9^/L), and received one chemotherapy cycle (AI: cytarabine, idarubicine) with ATRA and ATO (Supplementary Material Figure S2).

During the three study periods, recommendations for the prophylaxis of Pneumocystis jirovecii infection were constant. All patients received trimethoprim/sulfamethoxazole (TMP/SMX), while different medications were used as prevention of fungal infections throughout the centers of PPLLSG (mostly posaconazole, voriconazole, itraconazole, or fluconazole). From the second period onwards, prophylactic use of ciprofloxacin or levofloxacin was recommended in patients with neutropenia, but the decision in this matter was left to the discretion of the attending physician.

### 2.4 Statistical analysis

Variables that include age and leukocyte count at initial diagnosis, gender, FAB classification, CNS involvement, treatment risk groups and presence of genetic abnormalities were analyzed. Frequency of endpoints (ED15, ED15-42, TRD) were compared between the treatment protocols as well as in relation to the selected patients’ by the Chi2 test or the Fisher exact test in the case of too small numbers in the analyzed subgroups. Predictors of early death were explore using Cox regression model. Results for which *p* < 0.05 were considered statistically significant. Statistical analysis was performed in SPSS (Statistical Package for Social Science) version 29.0.

## 3 Results

### 3.1 Characteristics of patients died before 42 day of therapy

Thirty children died within 6 weeks of therapy (before day 42), 15 girls and 15 boys. The median age of those children was 10.7 years (1 day to 18 years). Most of the patients (*n* = 16, 53%) were diagnosed with acute monocytic leukemia (FAB M5). Among 46 patients with acute promyelocytic leukemia (APL, FAB M3) in the entire study, four children died before the 15th day of therapy. All four children were diagnosed in the first period of the study, when APL was treated with chemotherapy and ATRA. The four children of the 33 children with APL diagnosed in the first study period (12%) died from bleeding complications, mostly intracranial hemorrhage and DIC. A child with APL and hyperleukocytosis who had died before beginning therapy due to massive intracranial hemorrhage was not included in the final analysis due to insufficient clinical data. In two consecutive treatment protocols, no early deaths were reported among children with APL.

CNS involvement (CNS 3) was found in three out of 30 children who died early, two before day 15 of therapy. The median leukocyte count on the day of diagnosis was 127.91 × 10^9^/L (68.7–499 × 10^9^/L). Hyperleukocytosis was recognized in 12 out of 30 patients (40%), which counted for 10% of total number of children with initial leukocyte count ≥100 × 10^9^/L in the study (12 out of 124). In four children (13%) leukocyte count was more than 200 × 10^9^/L.

Two children with APL had more than 100 × 10^9^/L at the time of diagnosis. Five children with hyperleukocytosis had genetic rearrangements of known poor prognosis, such as: complex karyotype (1 child); t(10;11)(p12;q23)/KMT2A::MLL10 (2 children); (8,16)(p11.p13)/KATA6::CREBBP (1 child); t(6;9)(p23;q34)/DEK::NUP214 (1 child), however, none of the patients with hyperleukocytosis had FLT3-ITD mutation. In five children with hyperleukocytosis, genetic data was not available. Six out of 11 children with unfavorable genetic abnormalities in leukemic cells died after day 15 of therapy, mainly due to complications from infections (bacterial sepsis). Among children with unfavorable genetics there were 6 children (6/19, 31%) with KMT2A rearrangements.

The median time since the beginning of therapy and death was 11 days (range: 1–41). In patients with APL the median time of survival was 8 days (5–10 days). No patients died before starting AML therapy and no one, regardless of the type of AML, achieved CR before day 42 of therapy.

The percentage of deaths during the period of application of three consecutive AML-BFM protocols was 4.9% (19 children) vs. 5.3% (7 children) vs. 3.1% (4 children), respectively (*p* = 0.625). The lowest incidence of ED15 was in AML-BFM 2019 Recommendations (2.3%), and the highest in AML-BFM 2012 Registry protocol (4.6%) (*p* = 0.441). The incidence of ED15-42 was the lowest and comparable for both AML-BFM 2019 Recommendations and AML-BFM 2012 Registry protocols (0.7%). The incidence of ED15-42 in AML-BFM 2004 Interim protocol was 2.3%, almost as high as ED15 (2.6%) (Table 1). Tables 2, 3 presents the characteristics of patients with ED.

**Table 1:** Early and treatment related deaths rate in three consecutive protocols: AML-BFM 2004 Interim, AML-BFM 2012 Registry, and AML-BFM 2019 Recommendations.

	Total number of patients *n* = 646	AML-BFM- 2004 Interim *n* = 385	AML-BFM 2012 Registry *n* = 131	AML-BFM- 2019 Recommendations *n* = 130	*p*
	No. of pts (%)	No. of pts (%)	No. of pts (%)	No. of pts (%)	
ED15	19 (2.9)	10 (2.6)	6 (4.6)	3 (2.3)	0.441
ED15-42	11 (1.7)	9 (2.3)	1 (0.7)	1 (0.7)	0.472
Total ED	30 (4.6)	19 (4.9)	7 (5.3)	4 (3.1)	0.625
TRD	22 (3.4)	20 (5.1)	2 (1.5)	0	**0** **.** **002**

ED, early death; ED15, early death before 15 day; ED15-42, early death between 15 and 42 day; TRD, treatment related death.

Bold values represent statistical significance of those values (less than 0.05).

**Table 2:** Early and treatment related deaths according to analyzed features.

Feature	Total of patients *n* = 646	ED15 *n* = 19	*p*	ED15-42 *n* = 11	*P*	TRD *n* = 59	*P*
	No. of pts (%^a^)	No. of pts (%)		No. of pts (%)		No. of pts (%)	
Sex							
Female	343 (53)	9 (3.0)	0.967	6 (2.0)	0.763	12 (3.4)	0.519
Male	303 (47)	10 (2.9)		5 (1.5)		10 (3.3)	
Age							
<1 year	71 (11)	3 (4.2)	0.481	**4** (**5.6)**	**0**.**029**	1 (1.4)	0.337
1–9 years	252 (39)	5 (2.0)		**2** (**0.8)**		8 (3.1)	
>10 years	323 (50)	11 (3.4)		5 (1.5)		13 (4.0)	
FAB classification							
M0	36 (5)	0	**0**.**018**^b^	1 (2.8)	0.224	0	0.526
M1	79 (12)	2 (2.5)		0		2 (2.5)	
M2	173 (27)	2 (1.2)		1 (0.6)		7 (4.0)	
M3	46 (7)	**4** (**8.7)**		0		0	
M4	103 (16)	1 (1.0)		2 (1.9)		7 (6.7)	
M5	146 (23)	10 (6.8)		6 (4.1)		3 (2.0)	
M6	6 (1)	0		0		1 (16)	
M7	44 (7)	0		1 (2.3)		2 (4.5)	
ND	13 (2)	0		0		0	
Risk group							
SRG	167 (26)	1 (0.6)	0.068	0	0.280	4 (2.3)	0.103
IRG	93 (14)	1 (3.7)		0		0	
HRG	383 (60)	14 (1.1)		11 (2.9)		18 (4.7)	
ND	3	3		0		0	
WBC at diagnosis (×10^9^/L)							
<50	421 (68)	**5** (**1.2)**	**0**.**003**^b^	5 (1.2)	0.262	16 (3.8)	0.264
50–100	70 (12)	3 (4.3)		1 (1.4)		2 (2.8)	
>100	124 (20)	**8** (**6.5)**		4 (3.2)		2 (1.6)	
ND	30	3		1		2	
Genetic abnormalities							
Favorable genetics	143 (22)	4 (2.8)	0.411^b^	0		3 (2.0)	
Unfavorable genetics	167 (29)	5 (2.8)		6 (6.8)	0.061	9 (5.3)	0.505
Normal karyotype	146 (22)	1 (0.7)		2 (2.2)		3 (2.0)	
Other	115 (17)	1 (0.2)		0		4 (3.4)	
ND	73 (10)	8 (10.3)		3 (4.1)		3 (4.1)	

^a^ Distribution of variable categories.

^b^ The Fisher Exact test.

ED, early death; ED15, early death before 15 day; ED15-42, early death between 15 and 42 day; TRD, treatment related death; DRD, disease related death; SRG, standard risk group; IRG, intermediate risk group; HRG, high risk group; WBC, white blood cells; ND, no data; NS, nonsignificant.

Bold values represent statistical significance of those values (less than 0.05).

**Table 3:** Detailed information on patients with early death before day 42.

Patient no.	Protocol	Gender	Age at diagnosis (years)	AML subtype (FAB)	WBC (×10^9^/L) at diagnosis	ECOG at diagnosis	Genetics	Survival (days)	Couse of death
1	2004	M	15.2	M5	55	2	ND	10	DIC
2	2004	M	16.7	M0	143	2	Complex karyotype	26	Sepsis
3	2004	M	16.0	M1	371	**3**	ND	5	Leukostasis, CNS bleeding
4	2004	F	3.9	M3	150	1	PML::RARA	6	DIC, CNS bleeding
5	2004	M	1.1	M5	ND	ND	ND	15	Progression. NR
6	2004	F	16.6	M5	3.2	3	Normal	14	MOF, pulmonary oedema
7	2004	F	12.4	M3	40.4	3	PML::RARA	5	DIC
8	2004	M	1 day	M5	107	ND	ND	34	Sepsis
9	2004	F	3 ms	M5	142	2	KMT2A::MLL10	29	Pneumonia
10	2004	F	14.8	M4	5.4	ND	ND	25	Sepsis
11	2004	F	17.1	M4	6.4	1	ND	17	Progression
12	2004	F	18.0	M2	8.3	ND	ND	11	Bleeding
13	2004	M	4 days	M7	31	1	Normal	23	MOF
14	2004	M	13.4	M3	91.6	ND	PML::RARA	10	DIC, CNS bleeding
15	2004	F	13.4	M5	80	3	Complex karyotype FLT3-ITD	21	Fungal Pneumonia
16	2004	F	9.1	M5	402	4	KATA6::CREBBP	5	ALS, leucostasis, CNS bleeding
17	2004	M	3.3	M3	196	3	PML::RARA	10	DIC, CNS bleeding
18	2004	M	8.5	M5	434	4	ND	1	ALS leucostatsis CNS bleeding
19	2004	F	8.1	M2	3.4	ND	Monosomy 7	41	DAD
20	2012	M	7 ms	M5	??	ND	ND	2	ALS Leucostatsis
21	2012	F	9 ms	M5	5.78	1	KMT2A::MLL10	42	Sepsis
22	2012	F	4 ms	M1	45.4	3	KMT2A::MLLT1	6	Sepsis
23	2012	M	10.8	M4	198	ND	NPM1::MLF1	1	Leucostasis
24	2012	M	17.5	M5	65	2	KMT2A::MLL10	13	Leucostasis
25	2012	M	12.3	M5	169	2	KMT2A:: AFDN	2	Pulmonary leukostsis
27	2019	F	4.5	M2	?	210	ND	1	Lecostasis
28	2019	M	14.1	M5	183	4	DEK::NUP214	33	Leukostasis
29	2019	F	2 ms	M5	29.4	4	KMT2A::MLL10	4	MOF Metabolic acidosis
30	2019	F	13.6	M5	291	3	ND	12	Leucostasis

F, female; M, male; ms, months; DIC, disseminated intravascular coagulation; ALS, acute lysis syndrome; ECOG, performance status; DAD, diffuse alveolar damage; ND, no data.

### 3.2 Risk factors for ED

The analysis of risk factors for ED in the study patients did not reveal any differences in terms of gender, risk group, genetic abnormalities in leukemia blast cells or central nervous system involvement (*p* > 0.05). Twenty-three children who died early were classified as HRG. Only three children from SRG died between days 15 and 42. All were treated according to AML-BFM protocol, in which the stratification to the risk groups was based only on FAB and the response to therapy. Two of them had unfavorable genetic conditions (monosomy 7 and complex karyotype), however, in accordance with the assumptions of the therapeutic protocol used at first study period, which did not consider genetic results, the patients were qualified for SRG.

Four children with APL were classified as HRG due to the number of leukocytes greater than 10 × 10^9^/L. In three patients treated according to AML-BFM 2012 Registry or AML-BFM 2019 Recommendations classification, it was not feasible to classify risk groups due to the lack of the date of cytogenetic or molecular findings.

None of the patients who died before day 42 of therapy had favorable genetics, excluding patients with APL (in 3 of 4 PML::RARA mutations were identified, in one no cytogenetic or molecular studies were performed, the diagnosis of APL was made on the basis of the morphology of leukemic blasts only). Unfavorable genetics was identified in 11 of 30 patients who died early.

There were more ED15 and ED15-42 in children older than 10 years compared to younger children, although the differences were not statistically significant. However, in the group of children up to 1 year of age, ED 15-42 occurred significantly more often compared to children aged 1–9 years (5.6% vs. 0.8%, *p* = 0.029).

A significant correlation was also found between ED15 and high leukocyte count. Leukocyte count above 100 × 10^9^/L were associated with the highest ED15 rate (*p* < 0.002). A higher rate of ED was found in patients with APL. Too much data on ECOG status was missing to perform a reliable statistical analysis, although information on ECOG status is included in Table 3, which provides details of patients who died before day 42 of therapy.

In the univariate analysis only initial high leukocyte count >100 × 10^9^/L, was a significant predictor of early death (Hazard ratio, HR for ED15: 5.620; 95% Confidence Interval, CI: 1.838–17.179; *p* = 0.02; HR for ED0-42: 1.44, 95% CI: 1.84–9.86, *p* < 0.001). Neither age nor FAB type were associated with ED. The multivariate analysis due to small number of patients was not undertaken.

### 3.3 Cause of ED

In 19 patients, death occurred before day 15 of treatment, in 11 between days 15 and 42. The most common cause of death before day 15 was leukostasis (9 children out of 19, 47.3%) and bleeding (6 children, 31.5%) and between 15 and 42 days—infections, mainly bacterial sepsis (3 children out of 11, 27.2%).

### 3.4 Risk factors for TRD

In the total study cohort (646 children), there were 22 deaths due to treatment toxicity, which occurred between day 84 and day 341 of treatment (median: 148 days; 4.9 months). Most of these children were over 10 years old, with a comparable gender distribution (12 girls and 10 boys). The incidence of TRD decreased substantially over the years. In the first period when the AML-BFM 2004 Interim protocol was used, TRD reached 5.1% (22 children), while in the second period (AML-BFM-2012 Registry) only 2 children died (1.5%), and no child in CR died from toxicity in the last period of study, when AML-BFM 2019 Recommendations protocol was used. Differences in the TRD rate between the three study periods were statistically significant (*p* = 0.002). The overall TRD for the entire study period was 3.4%. Unfavorable genetics were detected more often in patients who died of treatment toxicities, but we didn't find any significant factor contributing to the higher incidence of TRD in our patient group. Table 2 shows the characteristics of patients with TRD.

### 3.5 Cause of TRD

The main cause of death were infections, mainly bacterial sepsis (10 children out of 22, 45.4%), fungal infections (3 children, 13.6%), and complications after HSCT in the first CR (7 children, 31.8%).

## 4 Discussion

Despite significant improvements in the treatment outcomes of AML patients, some of them still die before starting treatment or at an early stage of therapy, before achieving disease remission. Our analysis found further improvement in ED during the analyzed period of time (2004–2023), when three BFM therapeutic protocols were used. Over 19 years of the study, the total number of early deaths decreased. The ED rates during the first two treatment protocols used: AML-BFM 2004 Interim and AML-BFM 2012 Registry were 4.9% and 5.3%, respectively, and were comparable to the ED rates obtained in the AML-PPLLSG 98 protocol (5%) (6). In the last period of the study, when the 2019 AML-BFM Recommendations protocol was used, ED rates dropped to 3.1%, however the differences in ED rates between the three study periods were not statistically significant. The temporary increase in ED between the first and second periods (4.9% vs. 5.3%) cannot be explained by any change in chemotherapy intensity or by any relevant change in supportive guidelines, as these did not differ between the two phases of the study. The study also showed improvement in the ED15-42 rate in the second and third periods of the study (ED15-42, 0.7% for both). During the first 2 weeks of induction chemotherapy, a high rate of early deaths was still observed, especially in the second period of the study (ED15, 4.6%). We assume that the development of supportive treatment, including the use of antibiotics, antifungal drugs also for prophylactic purposes, primarily contributed to the improvement of ED15-42 rates, as infections were the main cause of ED15-42 in the first period of the study.

Similar trends of significant improvement in ED rates have also been reported by other study groups, such as BFM, NOPHO (Nordic Society of Pediatric Hematology and Oncology), DCOG (Dutch Childhood Oncology Group), COG (Children's Oncology Group), and St. Jude Research Hospital (2, 7–11). In the BFM studies, the overall number of early deaths decreased over time from 8.1% (1987–1992) to 2.2% (2011–2012), *p* = 0.001 (7). In the NOPHO trials conducted between 1984 and 2003, NOPHO-AML-84, 88 and −93 the deaths rose from 11% to 29%, respectively, but then decreased to 8% in NOPHO-AML-93 (8). Klein et al. also observed recent progress in reducing ED and TRD in children with AML treated by the Dutch Pediatric Oncology Group between 1998 and 2014. They showed that 12 of 245 children died early (4.9%). ED was 5.1% (*n* = 6) in the ANLL-97/MRC AML-12 study (1998–2002), 6.7% (*n* = 4) in the AML-15 study (2002–2009), and 3% (*n* = 2) in the DB AML-01 study (2009–2014), excluding deaths before treatment (*p* < 0.05) (11). Similarly, Alexander and colleagues from St. Jude Children's Research Hospital reported a significant decrease in the incidence of ED. The 5-year cumulative incidence of ED and TRD improved from 18.5% in the AML97 study (1997–2002) to 7.9% in the subsequent AML02 study (2002–2008). The lower incidence of ED and TRD contributed significantly to improved overall survival in the AML02 study (9).

APL is different from other types of acute myeloid leukemia. Over recent decades, the incidence of ED in adults and children has decreased significantly. The abandonment of intensive chemotherapy in most patients and the implementation of ATRA and arsenic acid together with intensive care measures, have significantly improved the results in APL. Before ATRA therapy, ED due to complications of coagulopathy occurred in up to 26% of patients. The current reported rate of ED is less than 15% (16–18). In our study, during the first period when ATRA was used concurrently with chemotherapy, the ED rate was 12%, while in the two other periods of the study when ATRA was used with ATO, no early deaths were reported. Children with APL in our study died from CNS bleeding and DIC as complications of the course of leukemia within 8 days after starting therapy. All recorded deaths among patients with APL occurred in the first period of the study, and no deaths were recorded in APL patients in subsequent study periods. We do not have a simple explanation for this, although in our opinion the acquired knowledge and experience of centers in managing patients, especially at the early stage of APL treatment, undoubtedly contributed to the reduction of early mortality.

In a large population-based analysis using data from the Surveillance, Epidemiology, and End Results (SEER) study from 1986 to 2015, the prevalence of ED in patients ≤18 years of age was 12%, with a decreasing prevalence over time (16% in 1986–1995; 13.2% in 1996–2005; and 10.1% in 2006–2015, respectively) (17). However, there was no information on the details of treatment in this large cohort of patients.

We also identified clinical parameters that influence early deaths in our study. Initial leukocyte count ≥100 × 10^9^/L, APL phenotype were associated with a higher ED rate before day 15, while age less than 1 year was associated with a higher ED rate between day 15 and 42. However, in the univariate analysis only initial high leukocyte count >100 × 10^9^/L, was a significant predictor of early death in our cohort of patients. Early mortality associated with hyperleukocytosis in patients with AML remains high, and many studies show that hyperleukocytosis is associated with worse outcomes (8, 19, 20). However, some studies do not show an effect of high leukocyte count on early mortality (11, 12). Hyperleukocytosis is detected in approximately 12%–22% of pediatric AML patients at diagnosis and leads to serious complications due to myeloblast leukostasis in the microcirculation of vital organs such as lungs, brain and kidneys, which causes hypoxia, thrombosis and bleeding. The high tumor burden associated with a large number of leukemic blasts also leads to another life-threatening situation, tumor lysis syndrome. Intensive supportive measures should be taken in this group of patients, especially in children with leukocyte counts above 200 × 10^9^/L (5). In our study, the total number of patients with hyperleukocytosis was comparable to other studies (124 children, 20%), and 10% of them (12 children) have died of leukostasis and its consequences, such as coagulopathy and MOF.

Different interventions are implemented to improve outcomes in patients with hiperleukocytosis. In comparison to hyperhydration, alkalization, cytoreductive therapy with cytarabine or hydroxyurea as methods of gradually reducing the number of myeloblasts in the circulation, leukapheresis, or exchange transfusions are much faster cytoreduction procedures. However, as Oberoi and colleagues showed in a systematic review, neither the leukapheresis strategy (*p =* 0.67) nor the hydroxyurea/low-dose chemotherapy (*p =* 0.23) do not reduce early mortality (19). Similarly, Chang and colleagues found no effect of cranial irradiation and leukapheresis on early death and intracranial hemorrhage in patients with hyperleukocytosis and AML (21). There are several meta-analyses of studies confirming that leukapheresis did not improve the treatment outcomes of AML patients with hyperleukocytosis. Adding to this the logistical and technical issues associated with this procedure, there are ultimately more arguments against the use of leukapheresis as a standard procedure for the treatment of hyperleukocytosis (19, 22–25). In our study, three of 12 (25%) patients with hyperleukocytosis underwent leukapheresis.

The concomitant use of multi-kinase FLT3 inhibitors, such as midostaurin, sorafenib (first generation) or gliteritinib (second generation) might be a therapeutic option in patients with hyperleukocytosis. Around 12% of patients with AML have FLT3-ITD mutations, while patients with hyperleukocytosis are characterised by a significantly higher rate of mutations ranging from 30% to 62% (26–30). That led Schmidt et al. to a retrospective analysis of four patients diagnosed with AML and hyperleukocytosis in a clinically critical state who were treated with one of the tyrosine kinase inhibitors. Sorafenib (multitargeted tyrosine kinase inhibitor that blocks the Fms-related tyrosine kinase 3 receptor in FLT3-ITD-mutated cells) was administered during the cytoreductive prephase, prior to starting induction therapy, before the FLT3-ITD status was known. Schmidt et al. reported four children with FLT3-ITD positive whose leukocyte counts dropped significantly to normal or below normal within 72 h of the first dose of Sorafenib. No serious drug toxicity was observed (31). Surprisingly, in our study only one patient of the 30 patients who died within 6 weeks of therapy presented FLT3-ITD mutations; however, some genetic data were missing in our cohort. Another strategy to quickly reduce high leukemic blast count could be the addition of bcl-2 inhibitors, such as venetoclax, to standard therapy in patients with hyperleukocytosis (32). The use of targeted therapies, FLT3 inhibitors or bcl-2 inhibitors in children and adolescents with hiperleukocytosis could be a treatment of choice; however, its role should be confirmed in prospective studies.

Patients with AML with or without hyperleukocytosis can have genetic aberrations other than the FLT3 mutation. A correlation between KMT2A rearrangement and the higher incidence of bleeding has been reported (15). Six children in our cohort 6/19, 31%) harbored KMT2A rearrangements, three of them died from infections, two patients from leukostasis complications, such as bleeding, one from MOF.

Another risk factor for ED in our study was the APL phenotype. All four patients with APL died from intracranial bleeding. The limited number of patients made the multivariable analysis impossible to study potential factors for ED in this group. Li et al. showed that older age (55 years) and lower socioeconomic status were independent risk factors for ED in a large cohort of pediatric and adult patients (17).

We also found a higher ED15-42 in infants compared to children aged 1–9 years (5.6% vs. 0.8%), *p* = 0.029. The main cause of death was infections. This is consistent with reports from other study groups (7–12). We hypothesize that younger age, known for a more aggressive course of AML, deeper aplasia associated with leukemia, greater susceptibility to treatment toxicity, combined with immaturity of the immune system, significantly influence the incidence of infections in this group of patients.

The leading cause of early mortality in our study cohort was leukostasis and bleeding directly related to leukemia with a median survival time of 11 days (range: 1–42) and 8 days (5–10 days) in patients with APL. This observation confirms that severe complications that result in early death are more often related to leukemia than chemotherapy and aplasia. Aplasia and infections were the frequent cause of death after 15 days of therapy, both ED15-42 and TRD. As reported by other study groups also in our study, the incidence of TRD gradually decreased during the study period, with no deaths in the first CR due to the toxicity of treatment (7). The highest number of TRD was observed in the first study period, mainly due to infections (5.1%). The same trend towards fewer deaths in CR was observed by the BFM group (7). The Dutch Oncology Group also showed a reduction in the cumulative incidence of TRD (5.9%, SE, 2.2% in 1998–2002 vs. 5%, SE, 2.8% in 2002–2009 vs. 4.6%, SE, 2.6% in 2009–2014), *p* > 0.05 (11).

Of note, the reduced rate of TRD reflects improvements in supportive care resulting, among other things, from the establishment of international guidelines such as the ECIL recommendations (33–35). Early referral to the intensive care unit (ICU) and systemic support are another key point in reducing early mortality in patients with AML, as reported by Mottal et al. (36). In the treatment of AML patients during induction therapy, close cooperation between the intensification specialist and the hematologist is crucial.

Although there is no clear consensus on the routine use of prophylactic antibiotics in patients with AML, there are studies showing a reduction in microbiologically confirmed infections and mortality (37, 38). There is a clear need for prospective, preferably randomized, studies to clarify the role of antibiotics in this area. In the second and third periods of the study, we observed a reduction in the incidence of ED15-42 (0.7% vs. 0.7% and 2.3% in the first period, respectively), which may have been related to improved supportive care measures, including prophylactic use antibiotics, as such recommendations appeared for the first time at that time. Unfortunately, accurate data on prophylactic antibiotic use in patients during the study period were not available.

Other research groups have also analyzed risk factors for ED. Similarly to our results, the NOPHO group found that the risk factors for ED were hyperleukocytosis and age <2 or 10 years (8). While the Dutch group showed that neither underweight nor hyperleukocytosis (≥100 × 10^9^/L at diagnosis), FAB type, CNS involvement or CBF (Core Bindig Factor) abnormalities were significantly associated with ED.

However, patients with APL were not included in the analysis (11). Gupta et al. also studied ED in low-income Central American countries and found that only a lower initial platelet count was an independent risk factor for ED (12). The risk of early mortality in elderly patients with newly diagnosed AML is higher compared to pediatric populations. In the study presented by Liu et al. 2-months mortality was 29.9%. Apart from elder age, ECOG ≥ 2, complex karyotype, bone marrow blasts above 70% and WBC ≥ 100 × 10^9^/L, and GFR < 45 were found independent predictor for early mortality (39).

Our study has several limitations. First, due to database limitations and the retrospective nature of our analysis, we were unable to obtain information on prophylactic antibiotic use, ECOG scores and results of genetic abnormalities in some patients. Second, the limited number of ED and TRDs explains the lack of statistical power of the reported results. Nevertheless, we demonstrate a continuous downward trend in ED and TRD in APL and non-APL patients over the past 19 years.

## 5 Conclusions

Adequate supportive care measurements are needed to reduce the rate of ED and TRD. The knowledge of ED predictors may help to improve supportive care strategies in children from the first days of therapies. Based on the results of our study, further reduction of ED should focus on interventions targeting bleeding and infections during induction therapy, including the development of detailed guidelines for patients with hiperleukocytosis. We want to emphasize that early implementation of cytoreductive therapy, the use of an ICU procedure in some patients, along with intensive hydration, platelets transfusions, are still crucial in the treatment of patients with AML. Rational antibacterial and antifungal prophylaxis, monitoring the occurrence of infections, and treating infections in patients with AML provide a chance to further improve TRD. More studies with a large cohort, conducted in a randomised setting, are needed to answer the question of how to further reduce ED and TRD in children with AML.

## Data Availability

The original contributions presented in the study are included in the article/Supplementary Material, further inquiries can be directed to the corresponding author.
