# Supplementary material for: Analysis of early and treatment related deaths among children and adolescents with acute myeloid leukemia in Poland: 2005–2023
Source: Front Pediatr. 2024 Oct 17;12:1482720. doi: 10.3389/fped.2024.1482720 (PMC11524810; doi:10.3389/fped.2024.1482720)
Supplement: Supplementary file 1 [file Datasheet1.pdf]

Supplementary Material

1 Supplementary Figures and Tables

1.1 Supplementary Figures

Supplementary Figure 1. The details of therapeutic regimens according to three consecutive study protocols: AML-BFM 2004 Interim and AML-BFM-2012 Registry and AML-BFM-2019 Recommendations protocols for non-APL patients

AML-BFM 2004 Interim

**SRG**	AIE	AI	hAM	HAE	Maintenance+ CNS RTX	
**HRG**	AIE	HAM	AI	hA	HAE	Allo-HSCT

AML-BFM 2012 Registry

**SRG inv(16)**	AIE	AI	hAM	HAE	Maintenance	
**SRG**	AIE	HAM	AI	hAM	HAE	Maintenance
**IRG**	AIE	HAM	AI/2-CDA	hAM	HAE	Maintenance
**HRG**	AIE	HAM	AI/2-CDA	hAM	Allo-HSCT	

AML-BFM 2019 Recommendations

**SRG inv(16)**	AIE	AI	hAM	HAE	Maintenance	
**SRG**	AIE	HAM	AI	hAM	HAE	Maintenance
**IRG**	AIE	HAM	AI	hAM	HAE	Maintenance
**HRG**	AIE	HAM	AI	hAM	Allo-HSCT	

Induction phases:

AIE: cytarabine 100 mg/m^2/day continuous infusion on days 1 and 2, followed by 30 min infusion every 12 h on days 3–8; idarubicin 12 mg/m^2/day, 30 min infusion days 3, 4, and 5; etoposide 150 mg/m2/day, 60 min infusion on days 6–8.

HAM: cytarabine 3 g/m^2/dose, 3 h infusion every 12 h on days 1–3 (6 doses); mitoxantrone 10 mg/m^2/day 30 min infusion on days 3 and 4.

Consolidation phases:

AI/2-CDA: cytarabine 500 mg/m^2/day 96-h infusion on days 1–4; idarubicin 12 mg/m^2/day, 30 min infusion on days 3 and 5; 2-chloro-2-deoxyadenosine 6 mg/m^2/day, 30 min infusion, on days 1 and 3.

AI: cytarabine 500 mg/m^2/day, 96-hours infusion on days 1–4; idarubicin 12 mg/m^2/day, 30 min infusion on days 3 and 5.

hAM: cytarabine 1 g/m^2/dose, 3 h infusion every 12 h on days 1–3 (6 doses); mitoxantrone 10 mg/m^2/day, 30 min infusion on days 3 and 4.

HAE: cytarabine 3 g/m^2/dose, 3 h infusion every 12 h on days 1–3 (6 doses); etoposide 125 mg/m^2/day, 60 min infusion on days 2–5.

Maintenance:

AML-BFM 2012: daily thioguanine 40 mg/m^2/day, orally; cytarabine 40 mg/m^2/day, i.v. or s.c., 4 days every 4 weeks for 1 year.

Supplementary Figure 2. The details of therapeutic regimens according to three consecutive study protocols: AML-BFM 2004 Interim and AML-BFM-2012 Registry and AML-BFM-2019 Recommendations protocols for APL patients

AML-BFM 2004 Interim

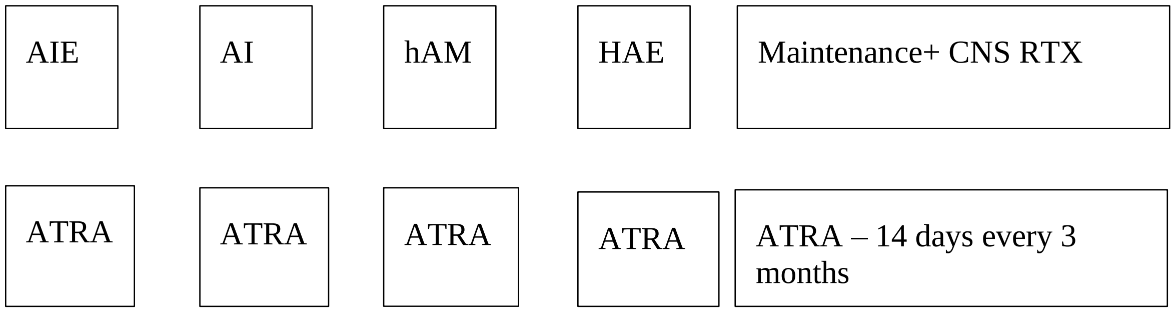

Induction phase: AIE: cytarabine 100 mg/m^2/day continuous infusion on days 1 and 2, followed by 30 min infusion every 12 h on days 3–8; idarubicin 12 mg/m^2/day, 30 min infusions days 3, 4, and 5; etoposide 150 mg/m^2/day, 60 min infusion on days 6–8.

Consolidation phases:AI: cytarabine 500 mg/m^2/day, 96-hours infusion on days 1–4; idarubicin 7.5 mg/m^2/day, 30 min infusion on days 3 and 5.

hAM: cytarabine 1 g/m^2/dose, 3 h infusion every 12 h on days 1–3 (6 doses); mitoxantrone 7 mg/m^2/day, 30 min infusion on days 3 and 4.

Intensifications phase: HAE: cytarabine 3 g/m^2/dose, 3 h infusion every 12 h on days 1–3 (6 doses); etoposide 125 m mg/m^2/day, 60 min infusion on days 2–5.

Maintenance: lasting 1 year: thioguanine 40 mg/m^2/day orally, cytarabine 40 mg/m^2/day intravenously for 4 consecutive days, every 4 weeks. ATRA—all trans-retinoid acid 25 mg/m^2/day for 14 days.

CNS, central nervous system.

AML-BFM-2012 Registry and AML-BFM-2019 protocols

Standard risk (WBC at diagnosis < 10x10^9/L)

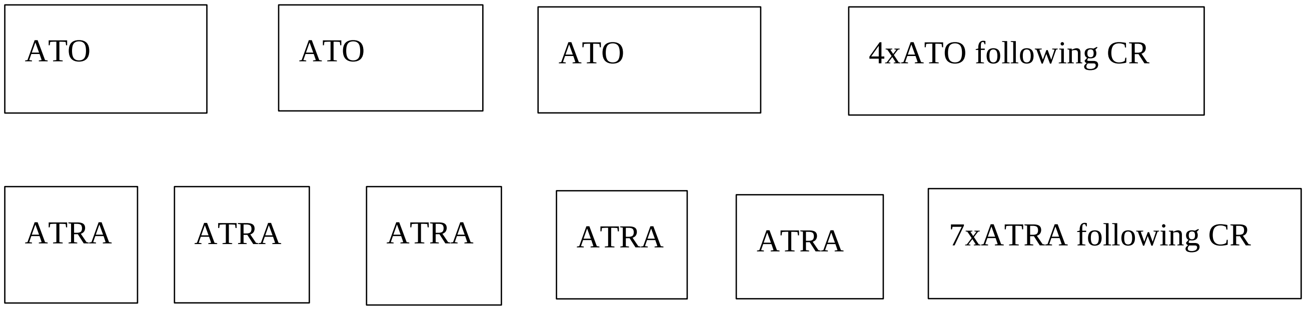

High risk (WBC at diagnosis ≥ 10x10^9/L)

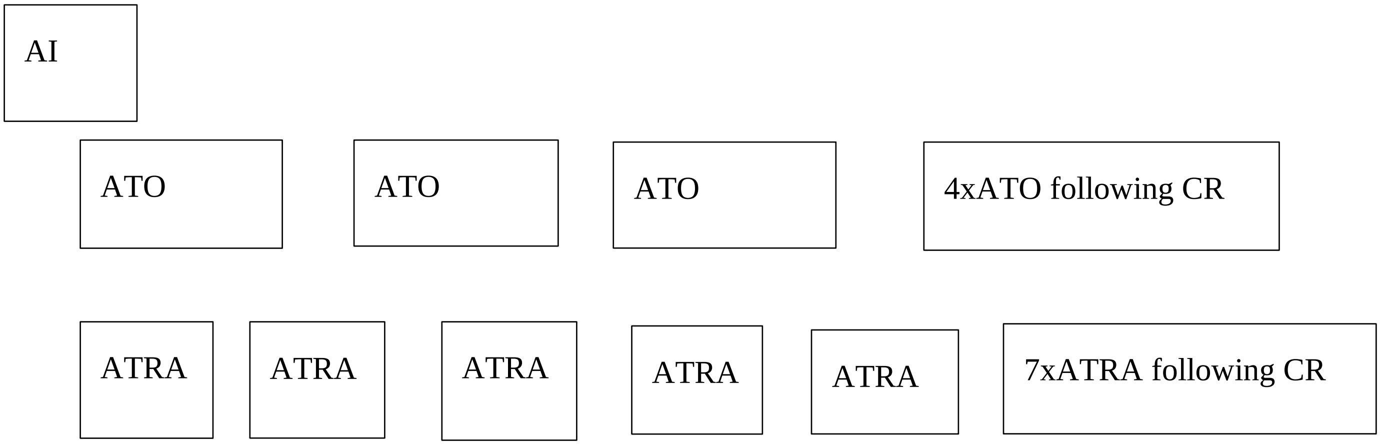

Induction phase: AI: cytarabine 100 mg/m^2/day, days 1–4, and cytarabine 100 mg/m^2 every 12 h, days 3-8, idarubicin 12 mg/m^2/day, 30 min infusion, days 3 and 5 and 7.

ATRA—all trans-retinoid acid 25 mg/m^2/day oral in two divided doses for 14 days.

ATO—arsentrioxide 0.15 mg/kg/day, starting at day 10 until morphologic CR, after 2 weeks brake: 4 cycles Monday–Friday 0.15 mg/kg/day i.v. for 4 weeks and 4 weeks break

1.2 Supplementary Table 1. Stratification to risk group according to treatment protocols

Protocol	AML-BFM-2004 Interim	AML-BFM 2012 Registry	AML-BFM 2019 Recommendations
**Standard risk group (SRG)**	M1/M2 with Auer rods [a,b]<br>AML with t(8;21)[a,b]<br>M4Eo with inv16[a,b]	t(8;21), inv(16), t(1;11),<br>NPM1, CEBPdm[c]	Inv(16)(p13.1q22)<br>t(16;16)(p13;q22)<br>t(8;21)(q22;q22)<br>t(1;11) (q21;q23)<br>Normal karyotype and NPM1-mutation<br>Normal karyotype and CEBPA (double mutation)c
**Intermediate risk group (IRG)**	Not applicable	All others[d]	All others[d]
**High risk group (HRG)**	M0 M1/M2 without<br>Auer rods<br>M4, M5, M6 and M7	t(4;11), t(5;11), t(6;11),<br>t(10;11), t(6;9), t(7;12),<br>der12p, isolated<br>monosomy 7, t(9;22),<br>FLT3-ITD and WT1mut,<br>complex karyotype	12p/ t(2;12), 5/5q,<br>FLT-ITD and WT1mut,<br>7 (not in combination with favorable/MLL-aberrations),<br>t(4;11)(q21;q23); KMT2A::AF4,<br>t(5;11)(q35.3;p15);NUP98::NSD1,<br>t(6;11)(q27;q23);KMT2A::AF6,<br>t(10;11)(p12;q23);KMT2A::AF10,<br>t(6;9)(p23;q34), t(7;12)(q36;p13),<br>t(9;22)(q34;q11), complex karyotype,<br>inv(3)(q21q26.2)/t(3;3)(q21;q26.2),<br>t(16;21)(p11;q22);FUS::ERG,<br>Inv(16)(p13.3q24.3);CBFA2T3::GLIS2

[a] In case of FLT3-ITD - reclassification to HRG;

[b] In case of BM blasts >5% on day 15 or blastic reconstitution between days 15 and 28—reclassification to HRG;

[c] In case of bone marrow blasts >20% on the days 21-28—reclassification to IRG;

[d] In case of bone marrow blasts > 20% on the days 21-28 or >5% on the days 42-56—reclassification to HRG
